# Clinical value of first-morning urine exfoliated cell HPV detection in the diagnosis of high-grade cervical lesions

**DOI:** 10.3389/fonc.2026.1878044

**Published:** 2026-07-08

**Authors:** Fang Li, Lihua Pei, Juan Lv, Mingfu Jiang, Hui Yang, Hailan Ma, Ning Zhou, Lingfei Li, Ying Luo, Zhengfu Wang, Haifeng Jiang, Na Zhao

**Affiliations:** 1 Pathology Department, Peking University First Hospital Ningxia Women and Children’s Hospital (Ningxia Hui Autonomous Region Maternal and Child Health Hospital), Yinchuan, China; 2 Pathology Department, Qingtongxia People’s Hospital, Qingtongxia, China; 3 Gynecological Department, General Hospital of Ningxia Medical University, Yinchuan, China; 4 Emergency Department, The First People’s Hospital of Yinchuan, Yinchuan, China; 5 Pathology Department, General Hospital of Ningxia Medical University, Yinchuan, China; 6 Gynecological Department, Peking University First Hospital Ningxia Women and Children’s Hospital (Ningxia Hui Autonomous Region Maternal and Child Health Hospital), Yinchuan, China

**Keywords:** cervical cancer screening, diagnostic accuracy, exfoliated cells, first-morning urine, high-grade cervical lesions, human papillomavirus

## Abstract

**Methods:**

This multicenter, cross-sectional diagnostic accuracy study enrolled 500 women attending gynaecology and colposcopy clinics at four institutions in Ningxia, China (June 2025–January 2026), using a disease-enriched design with predefined histopathological groups. All participants simultaneously underwent FMU HPV testing and cervical co-testing (liquid-based cytology [TCT] + HPV DNA). Those with abnormal co-testing underwent colposcopy and biopsy. Groups were defined histopathologically: cervical cancer (n=80), high-grade lesion/CIN2–3 (n=120), low-grade lesion/CIN1 (n=50), and no lesion (n=250).

**Results:**

FMU HPV detection rates were 96.25%, 86.67%, 58.00%, and 28.00% in the four groups (P<0.001). For CIN2+, FMU achieved sensitivity 90.50%, specificity 67.00%, PPV 64.64%, NPV 91.36%; traditional cervical testing achieved 93.00%, 64.67%, 63.70%, and 93.27%, with no significant differences (all P>0.05; no predefined non-inferiority margin was tested). Per-protocol compliance (97.20% vs 84.80%) and satisfaction (95.40% vs 79.60%) favoured FMU testing (both P<0.001); the compliance advantage persisted in an intention-to-treat analysis (76.2% vs 66.5%, P<0.001).

**Conclusions:**

In this disease-enriched diagnostic cohort, FMU HPV testing showed diagnostic performance comparable to that of traditional cervical HPV testing for CIN2+ detection, with no predefined non-inferiority margin tested, and offered substantially superior patient acceptability. These findings support FMU HPV testing as a promising complementary, non-invasive option that warrants validation in average-risk screening populations before routine implementation.

## Introduction

Cervical cancer is the fourth most common malignancy among women globally and a leading cause of cancer mortality in low- and middle-income countries (1). According to the most recent global estimates based on GLOBOCAN 2020, approximately 604,127 new cases and 341,831 deaths were attributed to cervical cancer in 2020, with approximately 90% occurring in low- and middle-income countries (2). In China, cervical cancer incidence has risen with a trend toward younger age at diagnosis; high-grade cervical lesions (CIN2/3) account for approximately 2–5% of all screened women (3). High-grade cervical intraepithelial neoplasia (CIN2/3) represents the critical precancerous stage preceding invasive carcinoma (4) and is frequently asymptomatic in early phases. Without timely intervention, an estimated 30–50% of untreated high-grade lesions progress to invasive cancer (5). The World Health Organization has set a 90–70–90 target for cervical cancer elimination, which requires sustained high-quality screening programs (6).

Current screening relies primarily on cervical cytology and HPV DNA testing, with colposcopy-directed biopsy for positive cases (7, 8). Conventional cervical specimen collection achieves high diagnostic accuracy (7) but is hampered by several barriers: it requires trained clinical personnel, necessitates intimate physical examination, and is declined by a substantial proportion of eligible women owing to psychological discomfort, religious or cultural barriers, or logistical constraints (8, 9). As a result, approximately 20–30% of age-eligible women do not participate in regular cervical cancer screening (8). Self-sampling strategies, including vaginal self-collection and urine-based testing, have been proposed as a means to overcome these barriers (9, 10).

Urine-based HPV detection has emerged as a promising non-invasive alternative. Cervical exfoliated cells shed passively into voided urine, making HPV DNA analysis feasible without clinical examination (11, 12). First-morning urine (FMU) contains a higher concentration of cervical-origin exfoliated cells than random void urine because the initial urinary stream flushes cells that have accumulated in the vaginal fornix overnight, thereby optimizing analytic sensitivity (13, 14). FMU collection is entirely home-based, requiring no healthcare personnel assistance. Prior systematic reviews and meta-analyses report that urine-based HPV testing shows good concordance with clinician-collected cervical specimens (14, 15), yet evidence on diagnostic performance stratified by lesion severity—and from multicenter Chinese settings—remains limited (9, 16).

This multicenter, cross-sectional study aimed to: (1) establish and optimize a standardized FMU HPV testing protocol suitable for use across diverse healthcare settings in Ningxia; (2) evaluate the diagnostic performance of FMU exfoliated cell HPV testing for CIN2+ detection relative to traditional cervical HPV testing using histopathology as the reference standard; (3) characterize HPV genotype distribution across lesion severity groups; and (4) assess participant compliance and satisfaction, thereby providing evidence to guide the integration of FMU HPV testing into cervical cancer screening programs.

## Methods

### Study design and participants

**Figure 1:**
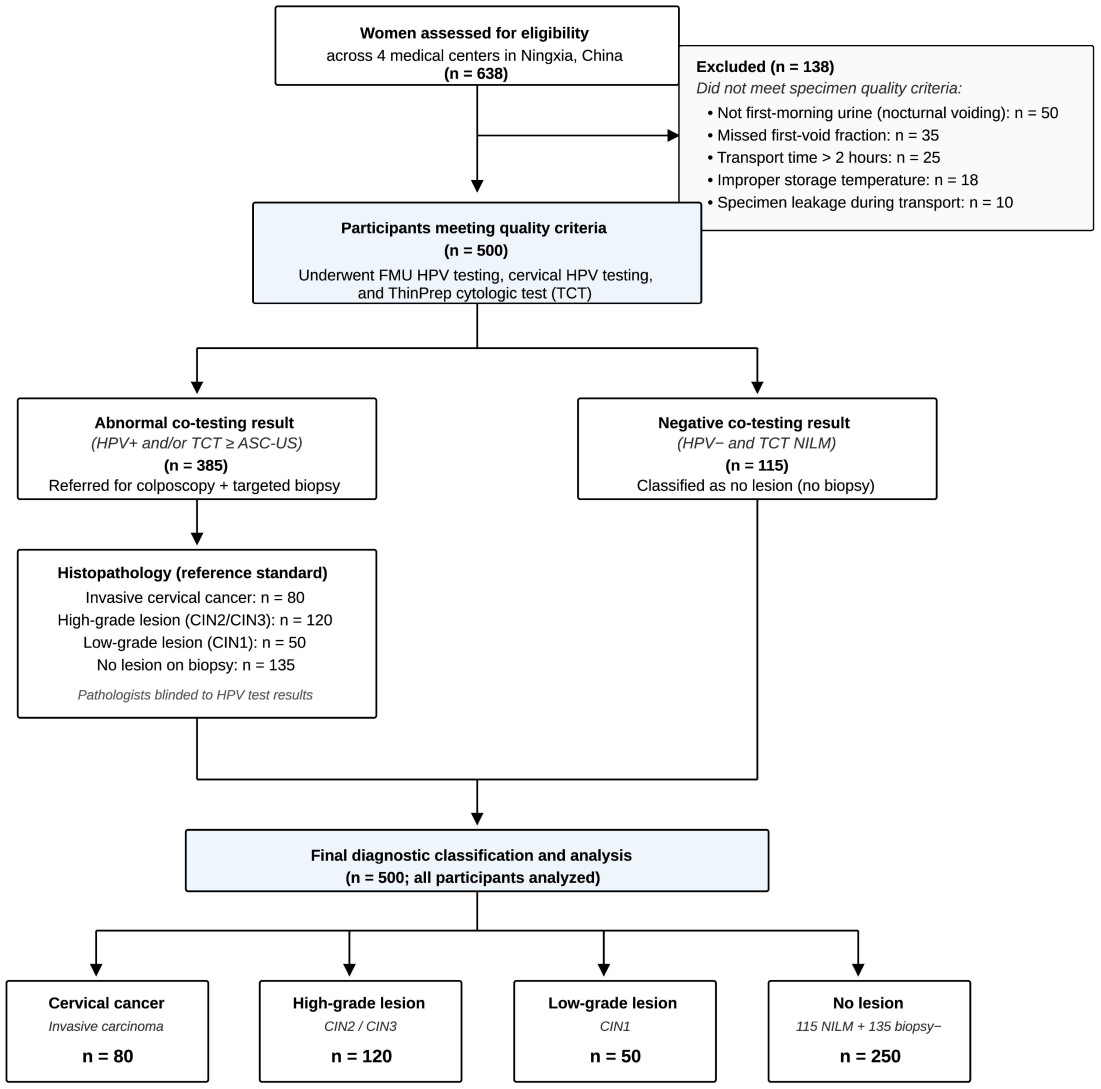
Study flow diagram. FMU, first-morning urine; TCT, ThinPrep cytologic test; ASC-US, atypical squamous cells of undetermined significance; CIN, cervical intraepithelial neoplasia; NILM, negative for intraepithelial lesion or malignancy.

This was a multicenter, cross-sectional diagnostic accuracy study. All participants were enrolled prospectively. The study was conducted between June 2025 and January 2026 across four medical institutions in Ningxia Hui Autonomous Region, China: Peking University First Hospital Ningxia Women and Children’s Hospital, General Hospital of Ningxia Medical University, Yongning County People’s Hospital, and Qingtongxia People’s Hospital. This multi-tier design—encompassing tertiary and secondary care institutions—was intended to ensure sample representativeness across geographic areas and healthcare levels in Ningxia. Study participant flow is illustrated in **Figure 1**. The study was approved by the Institutional Medical Ethics Committee of Peking University First Hospital Ningxia Women and Children’s Hospital (Approval No. KJ-LL-2024024) and by the ethics committees of all participating institutions. All participants provided written informed consent. The study was conducted in accordance with the Declaration of Helsinki.Recruitment pathway and study population. Participants were recruited from women attending gynaecology outpatient and colposcopy clinics at the participating centres, rather than from an unselected, average-risk, population-based screening cohort. To ensure a sufficient number of histologically confirmed lesions for diagnostic-accuracy estimation, the study used a disease-enriched design with enrolment into predefined diagnostic categories (cervical cancer, n=80; high-grade lesion/CIN2–3, n=120; low-grade lesion/CIN1, n=50; no lesion, n=250). Consequently, the CIN2+ prevalence in the analysed cohort (40%) reflects this enrichment by design and is substantially higher than the 2–5% expected in average-risk screening populations. This design supports robust head-to-head comparison of the two specimen types but limits direct extrapolation of prevalence-dependent indices (PPV and NPV) to routine screening settings, as discussed below.A total of 638 women were initially recruited. After applying the standardized specimen quality criteria, 138 participants were excluded for the following protocol-related reasons: not first-morning urine due to nocturnal voiding (n=50), failure to collect the first-void urine fraction (n=35), transport time exceeding 2 hours (n=25), improper storage temperature (n=18), and specimen leakage during transport (n=10). The remaining 500 participants met all quality requirements and were included in the final analysis, fulfilling the pre-specified sample size requirements.

### Inclusion and exclusion criteria

Inclusion criteria: (1) female sex; (2) age ≥18 years; (3) ability to provide FMU specimen; (4) complete clinical data. Exclusion criteria: (1) concurrent malignancy other than cervical cancer; (2) severe cardiac, hepatic, or renal dysfunction; (3) pregnancy or lactation; (4) urinary tract infection within the preceding 3 months; (5) autoimmune disease; (6) psychiatric disorder or cognitive impairment.

### Diagnostic classification

Participants were classified per histopathological diagnosis, in accordance with 《Zhonghua Fu Chan Ke Xue》 (17) and the 《Comprehensive Guidelines for Cervical Cancer Prevention and Control in China》 (18): (1) Cervical cancer: histopathologically confirmed invasive carcinoma (n=80); (2) High-grade cervical lesion: CIN2 or CIN3 (n=120); (3) Low-grade cervical lesion: CIN1 (n=50); (4) No lesion: negative intraepithelial lesion or malignancy on cytology, with absence of CIN or malignancy on biopsy where performed (n=250; including 115 with negative co-testing and 135 with abnormal co-testing but biopsy-confirmed absence of CIN or carcinoma).

### Quality control

A standardized “First-Morning Urine HPV Testing Standard Operating Procedure” was implemented across all centers, governing specimen collection, preservation, transport, and processing. All specimens were centrally analyzed at the Pathology Department of Peking University First Hospital Ningxia Women and Children’s Hospital by designated technicians using identical reagents and equipment, minimizing inter-site analytical variability. Each batch of testing included a negative control, a positive control, and an internal control (β-globin) to monitor contamination, reagent performance, and DNA extraction efficiency.

### Specimen collection and HPV DNA testing

First-morning urine (FMU) was defined as the first urine voided after waking, of which the initial (first-void) fraction was collected. Participants collected ≥30 mL of FMU within 1 hour of waking, including the initial 10-mL fraction of the urinary stream, into a sterile 50-mL tube (14). Samples were processed within 2 hours. For DNA extraction, urine was centrifuged at 3,000 rpm for 15 minutes; the pellet was resuspended in 1,000 μL RIPA lysis buffer, heat-treated at 95 °C for 10 minutes, and cooled to room temperature (12). Traditional cervical sampling: exfoliated cells were collected with a standardized cervical brush in the lithotomy position and placed in liquid preservation medium. HPV DNA genotyping: both specimen types were analyzed on the BOHUI Nucleic Acid Chip Detection System (Model BHF-VI) detecting 24 HPV genotypes: 16 high-risk (HPV 16, 18, 31, 33, 35, 39, 45, 51, 52, 53, 56, 58, 59, 66, 68, 73, 82, 83) and 6 low-risk (HPV 6, 11, 42, 43, 44, 81).

### Colposcopy and histopathological reference standard

Participants with HPV-positive results or cytological abnormalities (TCT ≥ ASC-US) underwent colposcopy with 3–5% acetic acid and Lugol’s iodine application. Targeted biopsies were obtained and histopathological diagnosis served as the reference standard, consistent with current clinical guidelines (19). Histopathologists were blinded to HPV test results.

### Compliance and satisfaction assessment

Compliance was defined as binary: “compliant” if all required testing steps were completed, “non-compliant” if any step was incomplete or refused. Satisfaction was assessed with a 5-point Likert scale (1=very dissatisfied to 5=very satisfied) covering convenience, privacy protection, physical comfort, wait time, and testing environment; a score ≥4 was considered satisfactory. The primary (per-protocol) denominator for compliance and satisfaction calculations was the 500 participants who met all specimen-quality criteria; the 138 women excluded for FMU specimen-quality non-conformance were withdrawn prior to any protocol-mandated traditional cervical HPV testing. Because these exclusions are directly related to FMU feasibility and could overestimate real-world acceptability, we additionally pre-specified an intention-to-treat (ITT) sensitivity analysis using the full recruited cohort (n=638) as the denominator (reported in Results and Discussion).

### Statistical analysis

Data were analyzed using SPSS 26.0 (IBM Corp., Armonk, NY). Continuous variables are presented as mean ± SD; between-group comparisons used independent t-test or one-way ANOVA with *post-hoc* LSD correction. Categorical variables are expressed as n(%) and compared by χ² or Fisher’s exact test. Diagnostic sensitivity and specificity were compared using McNemar’s test (paired design); PPV and NPV were compared using generalized estimating equations (GEE) to account for prevalence dependence and asymmetric distributions. All tests were two-tailed; P<0.05 was considered statistically significant.

Sample size justification. The required sample size was driven by the primary objective of estimating the sensitivity of FMU HPV testing for CIN2+ detection with adequate precision. Assuming an expected sensitivity of approximately 90%, a two-sided α of 0.05, and a desired absolute precision (margin of error) of ±8%, approximately 55 histologically confirmed CIN2+ cases were required. To permit stratified analysis across lesion-severity groups and paired comparison with traditional cervical testing at 80% statistical power, the target was increased to ≥150 CIN2+ cases. Allowing for an anticipated FMU invalid-specimen rate of approximately 20%, at least 600 women were planned for recruitment. The final analysed cohort (n=500, including 200 CIN2+ cases) satisfied these pre-specified requirements.

## Results

### Baseline characteristics

Baseline characteristics are presented in **Table 1**. The cervical cancer group had a significantly higher mean age (52.33 ± 8.29 years), menopausal rate (52.50%), and prior HPV infection history (56.25%) compared with other groups (all P<0.001).

**Table 1:** Baseline characteristics of participants in the four study groups.

Characteristic	Cervical cancer (n=80)	High-grade lesion (n=120)	Low-grade lesion (n=50)	No lesion (n=250)	P value
Age (years), mean ± SD	52.33 ± 8.29	47.66 ± 9.32	44.90 ± 8.33	44.59 ± 9.09	<0.001
Menopausal, n (%)	42 (52.50)	50 (41.67)	18 (36.00)	58 (23.20)	<0.001
Prior HPV history, n (%)	45 (56.25)	52 (43.33)	16 (32.00)	48 (19.20)	<0.001

SD, standard deviation; HPV, human papillomavirus. P values from one-way ANOVA (continuous) or χ² test (categorical).

### Study flow and participant allocation

The complete participant flow, including co-testing results, colposcopy referral pathway, and final group allocation, is depicted in **Figure 1**. Of the 500 enrolled women, 385 had abnormal co-testing and underwent colposcopy and biopsy; 115 had negative co-testing and were classified as no-lesion. All 500 participants were included in the final analysis.

### HPV detection rates across diagnostic groups

**Figure 2:**
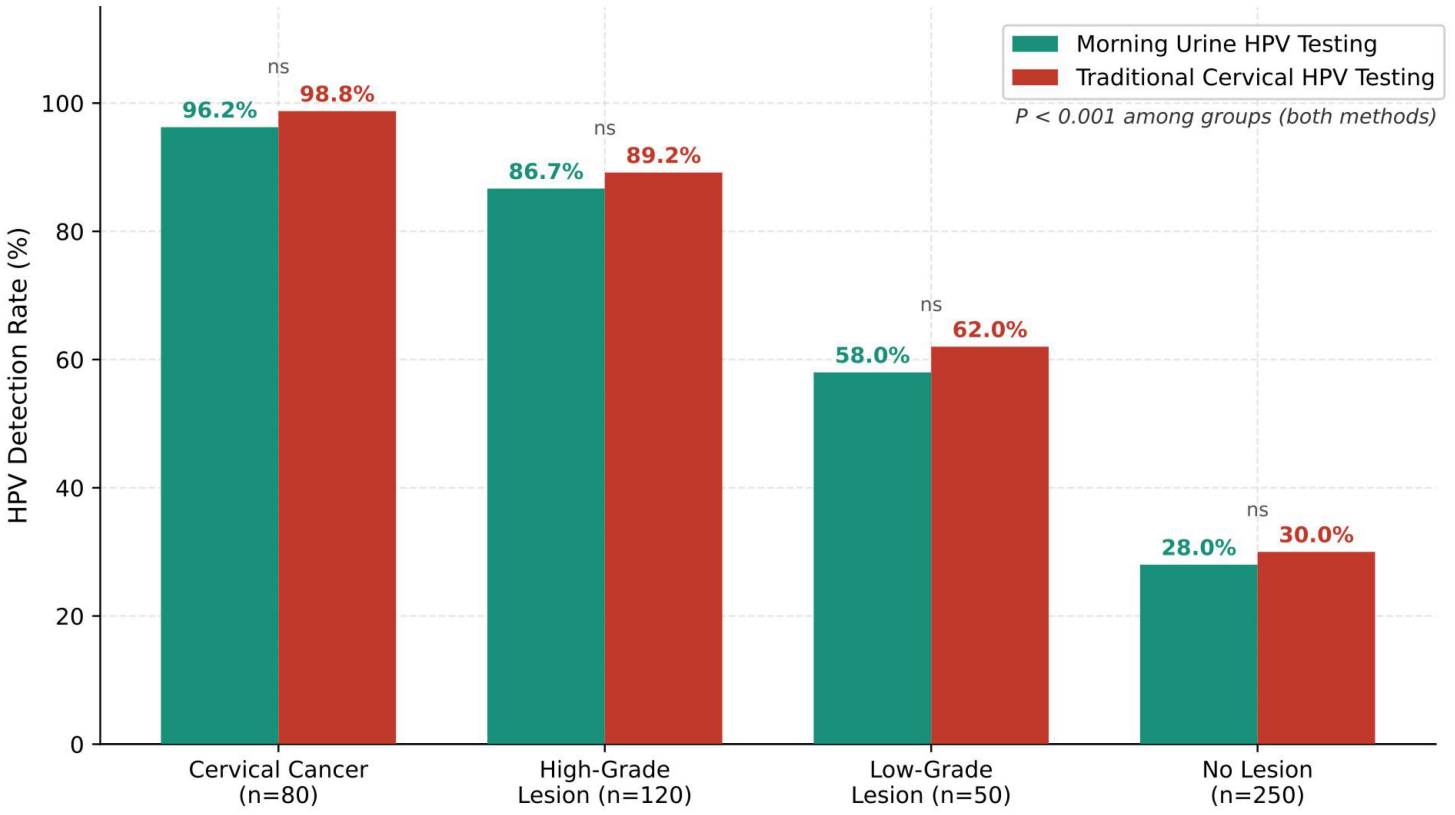
HPV detection rates across the four diagnostic groups. Morning urine HPV testing (teal) versus traditional cervical HPV testing (red). “ns” denotes no significant difference between methods within each group (McNemar’s test, P>0.05). P<0.001 for gradient among groups (both methods).

FMU HPV detection rates showed a significant gradient across groups: 96.25% (77/80) in cervical cancer, 86.67% (104/120) in high-grade lesions, 58.00% (29/50) in low-grade lesions, and 28.00% (70/250) in the no-lesion group (P<0.001 among groups). All FMU specimens successfully amplified the β-globin internal control, confirming adequate sample quality and successful DNA extraction. Traditional cervical HPV detection rates followed a parallel gradient: 98.75%, 89.17%, 62.00%, and 30.00%, respectively (P<0.001). No significant difference between the two methods was found within any single group (all P>0.05; **Table 2**; **Figure 2**).

**Table 2:** HPV detection rates in the four diagnostic groups, n (%).

Group	n	Morning urine HPV+, n (%)	Traditional cervical HPV+, n (%)	P value^a^
Cervical cancer	80	77 (96.25)	79 (98.75)	0.617
High-grade lesion (CIN2/3)	120	104 (86.67)	107 (89.17)	0.531
Low-grade lesion (CIN1)	50	29 (58.00)	31 (62.00)	0.678
No lesion	250	70 (28.00)	75 (30.00)	0.604
P value^b^ (among groups)		<0.001	<0.001	

^a^McNemar’s test (between methods within each group). ^b^χ² test (among four groups).

### HPV genotype distribution

Among HPV-positive participants, HPV16 remained the most prevalent genotype in both specimen types. In FMU specimens, HPV16 detection rates were 62.34% (48/77) in the cervical cancer group, 54.81% (57/104) in high-grade lesions, 44.83% (13/29) in low-grade lesions, and 27.14% (19/70) in the no-lesion group. Corresponding HPV18 rates were 28.57%, 21.15%, 13.79%, and 10.00%. Both HPV16 and HPV18 rates were significantly higher in the cancer and high-grade groups than in the low-grade and no-lesion groups (P<0.05) (20, 21). Additional high-risk genotypes also showed noteworthy distributions across groups. In FMU specimens, HPV58 reached 17.24% (5/29) in low-grade lesions, whereas HPV52, HPV33, HPV31, and the pooled Other HR-HPV category were consistently detected in cervical cancer and high-grade lesion groups (**Table 3**).

**Table 3:** HPV genotype distribution among HPV-positive participants, n (%).

A. HPV genotype distribution among HPV-positive participants — traditional cervical specimens, n (%)									
Group	HPV+, n	HPV16, n(%)	HPV18, n (%)	HPV52, n (%)	HPV58, n(%)	HPV33, n (%)	HPV31, n(%)	Other HR-HPV, n (%)
Cervical cancer	79	50 (63.3)	23 (29.1)	2 (2.5)	4 (5.1)	4 (5.1)	0 (0.0)	35 (44.3)
High-grade lesion	107	60 (56.1)	24 (22.4)	9 (8.4)	5 (4.7)	0 (0.0)	3 (2.8)	39 (36.4)
Low-grade lesion	31	14 (45.2)	5 (16.1)	1 (3.2)	3 (9.7)	4 (12.9)	0 (0.0)	12 (38.7)
No lesion	75	22 (29.3)	8 (10.7)	4 (5.3)	6 (8.0)	6 (8.0)	2 (2.7)	43 (57.3)
P value^a^ (among groups)		<0.05	<0.05	NS	<0.05	<0.05	NS	<0.05

B. HPV genotype distribution among HPV-positive participants — morning urine (FMU) specimens, n (%)									
Group	HPV+, n	HPV16, n(%)	HPV18, n (%)	HPV52, n (%)	HPV58, n(%)	HPV33, n (%)	HPV31, n(%)	Other HR-HPV, n (%)
Cervical cancer	77	48 (62.3)	22 (28.6)	3 (3.9)	3 (3.9)	4 (5.2)	4 (5.2)	34 (44.2)
High-grade lesion	104	57 (54.8)	22 (21.2)	3 (2.9)	4 (3.8)	5 (4.8)	6 (5.8)	40 (38.5)
Low-grade lesion	29	13 (44.8)	4 (13.8)	2 (6.9)	5 (17.2)	0 (0.0)	0 (0.0)	14 (48.3)
No lesion	70	19 (27.1)	7 (10.0)	4 (5.7)	5 (7.1)	4 (5.7)	5 (7.1)	43 (61.4)
P value^a^ (among groups)		<0.05	<0.05	<0.05	<0.05	<0.05	<0.05	<0.05

HR-HPV, high-risk human papillomavirus. Some subjects had co-infections; percentages may exceed 100%. NS, not significant (P≥0.05). ªP value from χ² test among four groups.

Multiple-genotype (co-)infections were common. Among FMU HPV-positive participants, 132/280 (47.1%) carried two or more genotypes, and 109/132 (82.6%) of these co-infections included HPV16 and/or HPV18; corresponding figures for traditional cervical specimens were 130/292 (44.5%) and 103/130 (79.2%). Overall, HPV16/18 was present in 164/280 (58.6%) of FMU-positive and 177/292 (60.6%) of traditional-positive participants, whereas 66/280 (23.6%) and 66/292 (22.6%), respectively, were positive for non-16/18 high-risk genotypes only. Within CIN2+ cases, 24/181 (13.3%) FMU-positive and 26/186 (14.0%) traditional-positive participants harboured only non-16/18 high-risk genotypes. Thus, although the majority of non-16/18 high-risk detections co-occurred with HPV16/18, a clinically meaningful minority of high-grade cases were attributable to non-16/18 high-risk genotypes alone.

### Diagnostic performance for CIN2+ detection

**Figure 3:**
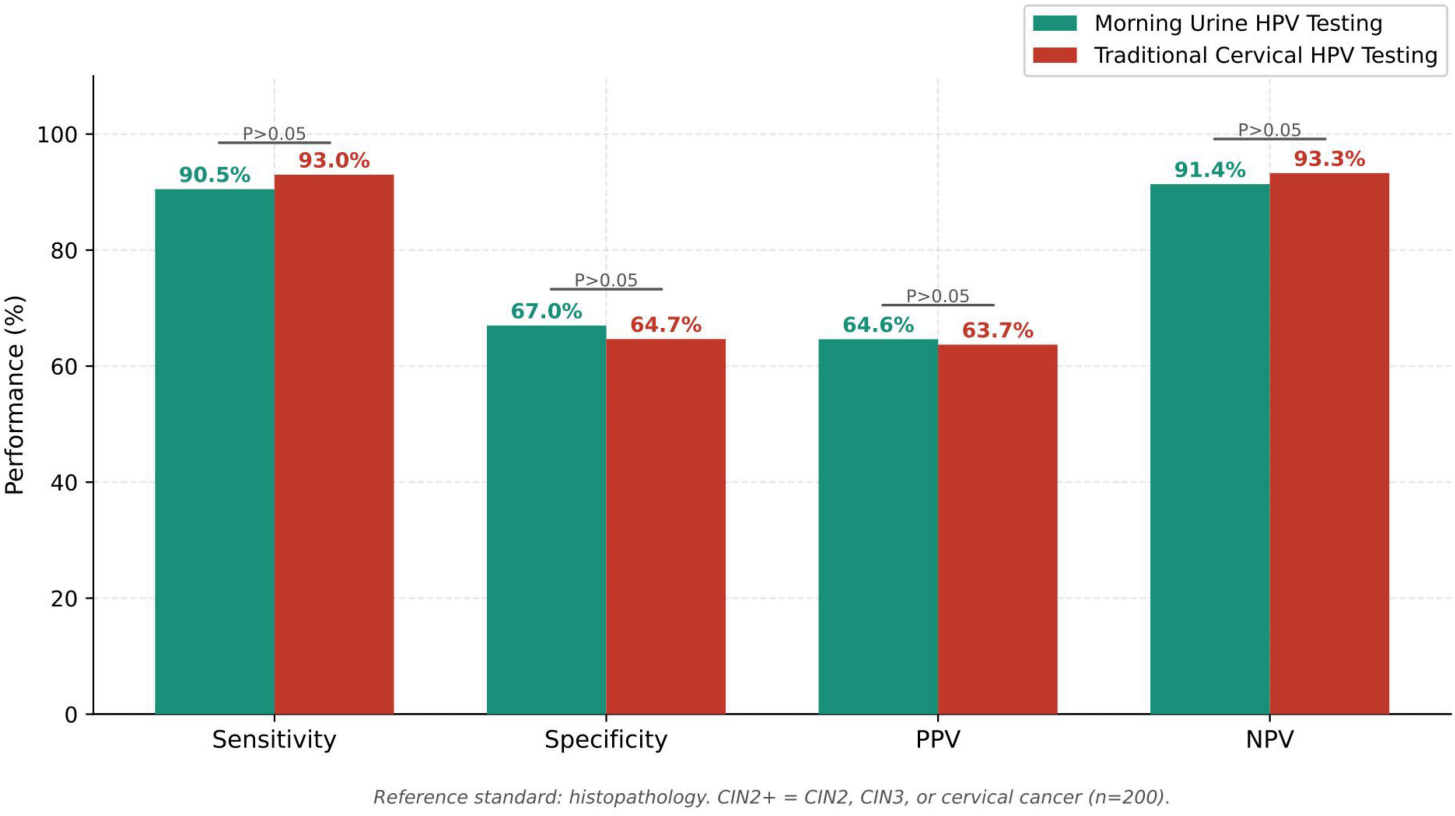
Comparison of diagnostic performance indices (sensitivity, specificity, PPV, NPV) for CIN2+ detection between morning urine HPV testing (teal) and traditional cervical HPV testing (red). Horizontal lines indicate pairwise comparisons; all P>0.05. Reference standard: histopathology. PPV, positive predictive value; NPV, negative predictive value.

Using histopathology as the reference standard (CIN2+ n=200; non-CIN2+ n=300), the diagnostic performance of both methods is shown in **Table 4** and **Figure 3**. FMU HPV testing yielded TP = 181, FN = 19, FP = 99, TN = 201, corresponding to sensitivity 90.50%, specificity 67.00%, PPV 64.64%, and NPV 91.36%. Traditional cervical HPV testing yielded TP = 186, FN = 14, FP = 106, TN = 194, corresponding to sensitivity 93.00%, specificity 64.67%, PPV 63.70%, and NPV 93.27%. No statistically significant difference was found for any performance index (all P>0.05), indicating comparable diagnostic performance between the two methods; because no predefined equivalence or non-inferiority margin was specified, these data should be interpreted as showing comparable—rather than formally equivalent—performance (14, 15).

**Table 4:** Diagnostic performance of morning urine vs traditional cervical HPV testing for CIN2+ detection.

Parameter	Morning urine HPV testing	Traditional cervical HPV testing	P value
True positives (TP)	181	186	—
False negatives (FN)	19	14	—
False positives (FP)	99	106	—
True negatives (TN)	201	194	—
Sensitivity, %	90.50	93.00	0.289^a^
Specificity, %	67.00	64.67	0.413^a^
PPV, %	64.64	63.70	0.751^b^
NPV, %	91.36	93.27	0.318^b^

PPV, positive predictive value; NPV, negative predictive value; CIN2+, CIN2, CIN3, or cervical cancer.

CIN2+ n=200; non-CIN2+ n=300. ^a^McNemar’s test. ^b^GEE model.

Concordance of false-negative results was examined among the 200 CIN2+ cases. FMU HPV testing was negative in 19 CIN2+ cases and traditional cervical testing in 14. All 14 traditional-negative CIN2+ cases were also FMU-negative (concordant double-negatives), and no CIN2+ case was traditional-negative while FMU-positive; the 5 additional FMU false negatives were traditional-positive, indicating cases in which cervical sampling detected high-risk HPV that FMU did not. Because tissue-based HPV genotyping of biopsy specimens was not performed, we could not determine whether the 14 concordant double-negative CIN2+ lesions were truly HPV-independent (or carried virus below the detection threshold) or whether high-risk HPV was present in the lesion but undetected by both liquid specimens.

### Participant compliance and satisfaction

**Figure 4:**
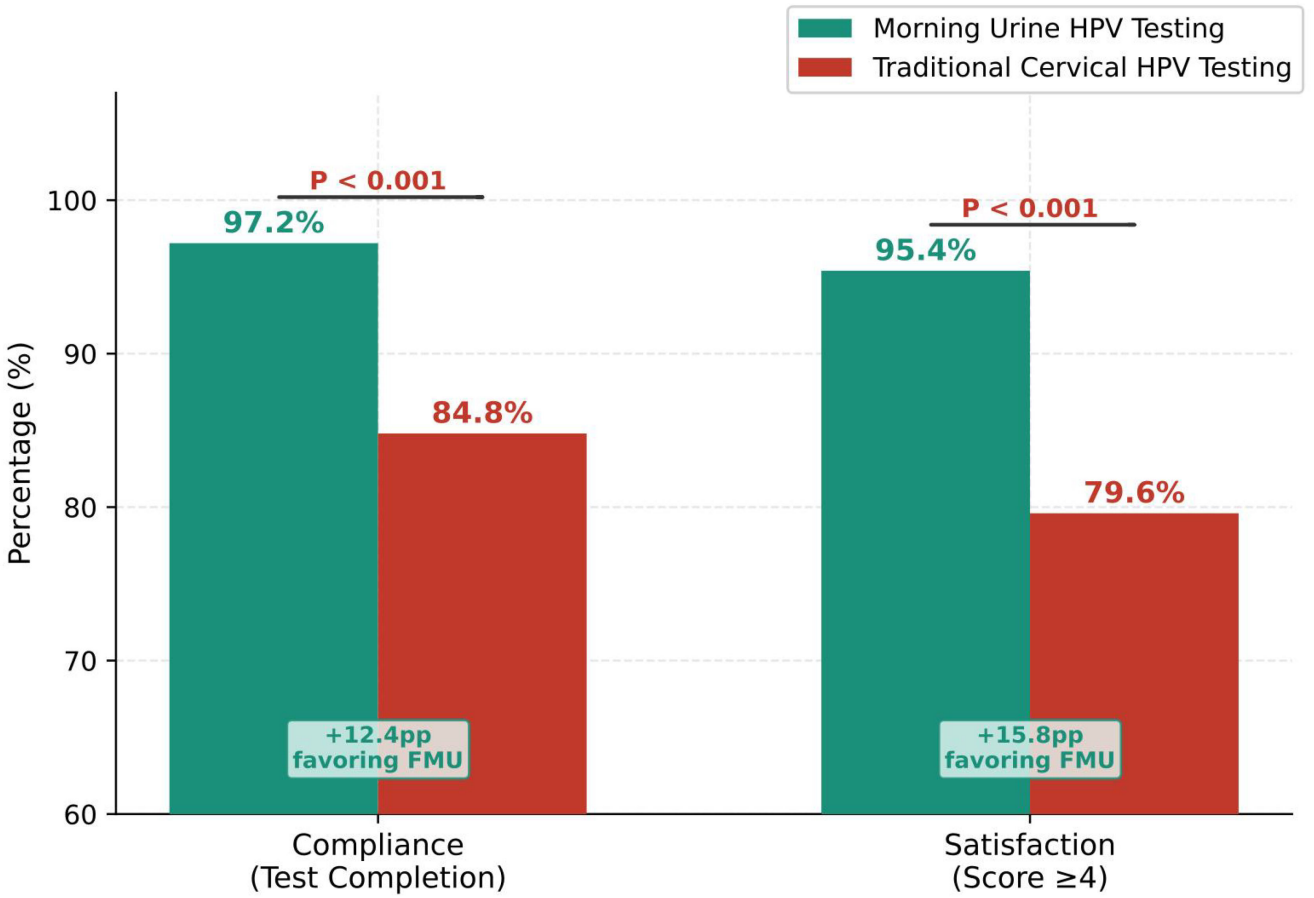
Participant compliance and satisfaction for morning urine HPV testing (teal) vs traditional cervical HPV testing (red). Percentage-point (pp) differences favoring FMU testing are indicated. P<0.001 for both comparisons.

Per-protocol compliance with FMU testing (97.20%, 486/500) was significantly higher than with traditional cervical testing (84.80%, 424/500; P<0.001). Per-protocol satisfaction (≥4 on the Likert scale) was also significantly higher for FMU testing (95.40%, 477/500) than traditional testing (79.60%, 398/500; P<0.001). To avoid overestimating real-world acceptability through exclusion of the 138 women with invalid FMU specimens, we additionally performed an intention-to-treat (ITT) analysis using the full recruited cohort (n=638) as the denominator, conservatively counting all excluded women as non-compliant for both methods; FMU compliance remained higher than traditional testing (76.2%, 486/638 vs 66.5%, 424/638; difference +9.7 percentage points, P<0.001). Both per-protocol and ITT results are presented in **Table 5** and **Figure 4**.

**Table 5:** Participant compliance and satisfaction with the two testing methods, n (%).

Metric	Morning urine HPV testing (n=500)	Traditional cervical HPV testing (n=500)	P value
Compliance, per-protocol (n=500), n (%)	486 (97.20)	424 (84.80)	<0.001
Satisfaction, per-protocol (n=500), n (%)	477 (95.40)	398 (79.60)	<0.001
Compliance, intention-to-treat (n=638), n (%)	486 (76.20)	424 (66.50)	<0.001

Compliance: completion of all required testing steps. Satisfaction: 5-point Likert scale (≥4 = satisfactory). P values: χ² test. Per-protocol denominator n=500; intention-to-treat (ITT) denominator n=638 (all recruited women, with the 138 excluded for invalid FMU specimens counted as non-compliant for both methods).

### Area under the ROC curve (AUC) for CIN2+ detection

**Figure 5:**
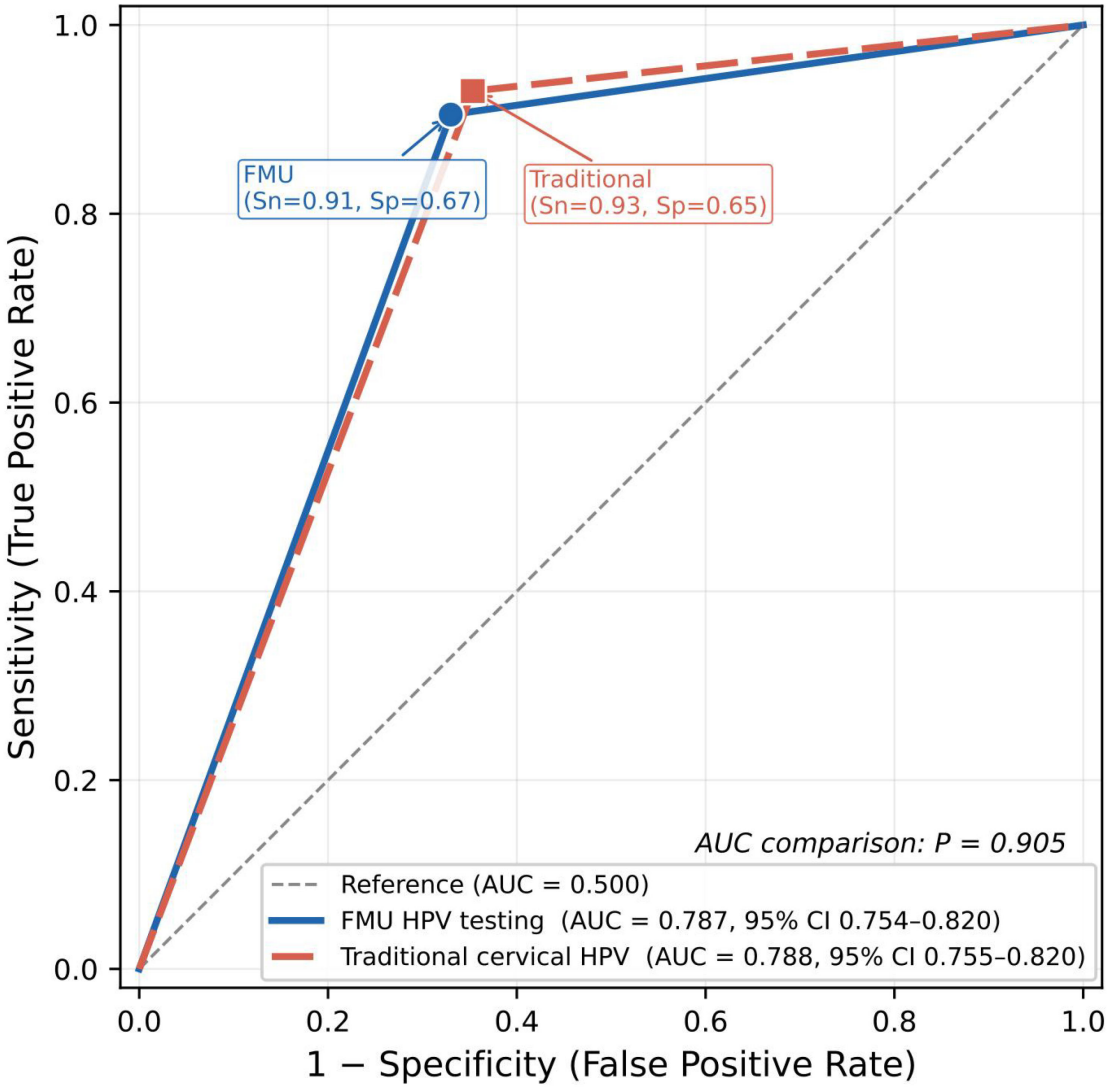
ROC curves for CIN2+ detection using FMU HPV testing (blue) and traditional cervical HPV testing (red). Diagonal dashed line represents the reference line. AUC values with 95% confidence intervals are shown in the legend. P = 0.887 for AUC comparison (bootstrap DeLong method).

The area under the receiver operating characteristic (ROC) curve (AUC) for detecting CIN2+ was 0.788 (95% CI: 0.753–0.820) for FMU HPV testing and 0.788 (95% CI: 0.755–0.820) for traditional cervical HPV testing. No statistically significant difference was found between the two AUC values (difference: −0.001, P = 0.887; **Table 6**; **Figure 5**). Both methods demonstrated comparable overall discriminative ability for CIN2+ detection.

**Table 6:** AUC comparison for CIN2+ detection.

Method	AUC	95% CI	Sensitivity (%)	Specificity (%)	P value (vs. FMU)
FMU HPV testing	0.788	0.753–0.820	90.50	67.00	—
Traditional cervical HPV	0.788	0.755–0.820	93.00	64.67	0.887

AUC, area under the ROC curve; CI, confidence interval; FMU, first-morning urine. P value from bootstrap DeLong method (2000 resamples).

## Discussion

This multicenter study systematically characterized the clinical performance of a standardized FMU HPV testing protocol across four institutions in Ningxia, China, with simultaneous capture of patient-reported compliance and satisfaction outcomes. Our findings demonstrate that FMU HPV testing achieves diagnostic performance comparable to that of traditional cervical HPV testing for CIN2+ detection—although no predefined equivalence or non-inferiority margin was tested—while offering substantially superior participant acceptability.

The graded pattern of HPV positivity across the four groups—from 96.25% in cervical cancer to 28.00% in the no-lesion group—reflects the established causal relationship between persistent high-risk HPV infection and cervical carcinogenesis (4). Virtually all invasive cervical cancers harbor HPV DNA, with HPV16 and HPV18 accounting for approximately 70% of cases globally (20). In our cohort, HPV16 detection rates in FMU specimens were 62.34% in the cervical cancer group and 54.81% in high-grade lesions, consistent with published data on HPV16 predominance in high-grade disease in East Asian populations (21, 22). This biological concordance validates FMU as a specimen type faithfully reflecting true cervical HPV carriage. Beyond HPV16 and HPV18, other high-risk genotypes also exhibited notable prevalence in our cohort. HPV58 was detected in 17.2% of FMU-positive low-grade lesion cases, suggesting its role in early-stage cervical disease in this region. HPV52 was found in 3.9% and 2.9% of HPV-positive cancer and high-grade lesion cases, respectively. HPV33 and HPV31 were present at 4.8–5.2% in high-grade and cancer groups by FMU testing. Notably, the “Other HR-HPV” category (encompassing HPV35, HPV39, HPV45, HPV56, HPV68, and related genotypes) accounted for 38.5–44.2% of HPV-positive cases in cancer and high-grade groups, underscoring that a substantial proportion of high-grade cervical disease in Ningxia is attributable to non-16/18 high-risk genotypes. These findings highlight the importance of comprehensive high-risk HPV genotyping beyond HPV16/18 for accurate risk stratification in this population. Notably, most non-16/18 high-risk detections co-occurred with HPV16/18 (109/132, 82.6% of FMU co-infections involved HPV16 and/or HPV18); nonetheless, 13.3% (24/181) of FMU-positive CIN2+ cases harboured only non-16/18 high-risk genotypes, indicating that a clinically meaningful minority of high-grade disease in this region would be missed by HPV16/18 genotyping alone and supporting comprehensive high-risk genotyping for accurate risk stratification.

During overnight recumbency, exfoliated cervical and vaginal cells accumulate in the vaginal fornix. The initial stream of first-morning urine flushes these cells into the voided specimen, making the first-void fraction optimal for HPV detection (12). Extended overnight accumulation concentrates these cells compared with random void urine (15). Collection of the initial urinary fraction—the first-void portion—maximizes exfoliated cell yield and HPV detection sensitivity, as confirmed by prior systematic reviews (14). Self-sampling based on this rationale has also been applied to vaginal specimens, where home-based collection has been shown to significantly improve participation rates in never-screened and underscreened populations (10, 23).

The diagnostic performance of FMU HPV testing (sensitivity 90.50%, specificity 67.00%, NPV 91.36%) aligns with prior meta-analyses comparing urine-based and clinician-collected HPV tests (14, 15). The high NPV (>91%) is particularly valuable in primary screening: a negative FMU HPV result carries high confidence for excluding CIN2+, supporting its use as a triage tool (16). The modest specificity (approximately 64–67%) of both methods reflects the known limitation of HPV testing as a standalone screen—a transient, self-clearing HPV infection does not necessarily represent progressive disease (3, 8). Song and Wang’s comparative study confirmed comparable sensitivity and specificity between urine and cervical HPV detection (24), and Yang et al. highlighted the potential for urine-based testing to expand screening access in underserved populations (25). The efficacy of HPV-based screening for preventing invasive cervical cancer has been established in large European randomized trials (7), providing a strong rationale for expanding HPV testing modalities.

Because predictive values depend on disease prevalence, the PPV and NPV observed here must be interpreted in the context of this disease-enriched cohort (CIN2+ prevalence 40%). In an average-risk screening population, where CIN2+ prevalence is typically 2–5%, the PPV of FMU HPV testing would be substantially lower and the NPV correspondingly higher than the values reported here. Sensitivity and specificity are comparatively less prevalence-dependent, but the clinical utility and predictive performance of FMU HPV testing in low-risk, asymptomatic, population-based screening settings remain uncertain and require dedicated validation. Accordingly, the present findings should not be extrapolated directly to routine population-based screening.

The most clinically compelling finding is the markedly superior participant acceptability of FMU testing. Compliance improved by 12.4 percentage points (97.20% vs 84.80%), and satisfaction by 15.8 percentage points (95.40% vs 79.60%), both highly significant (P<0.001). Given that population-level cervical cancer mortality reduction depends principally on screening coverage rather than marginal performance differences (6), this improvement in participation has potentially greater public health impact than small differences in test sensitivity. The higher satisfaction reflects the perceived benefits of FMU testing: non-invasiveness, home-based self-collection, privacy, and no gynecological examination—factors particularly relevant for unmarried women, elderly women, and those with cultural, religious, or logistical barriers (8, 9). Home-based self-sampling has previously been shown to substantially improve participation rates in large randomized trials (23), and in Ningxia—characterized by geographic remoteness, ethnic diversity, and variable healthcare infrastructure—these advantages are especially consequential. As a sensitivity analysis, applying an intention-to-treat framework (denominator n=638, treating all 138 excluded participants as non-compliant for both methods), FMU compliance remained 76.2% (486/638) versus 66.5% (424/638) for traditional cervical testing—a difference of +9.7 percentage points (P<0.001 by chi-square). Even under this most conservative assumption, the compliance advantage of FMU testing is statistically robust, confirming that the compliance superiority of morning urine testing is not an artefact of the per-protocol exclusions.

This study additionally demonstrates the feasibility of standardized FMU HPV testing across a hierarchically diverse multi-institution network (tertiary to county-level hospitals). The centralized laboratory model minimized inter-site analytical variability and provides a quality-assurance framework applicable to real-world scale-up (26). Combined with the high-risk HPV testing efficacy established in the Chinese screening context (3), these findings support further evaluation of FMU HPV testing as a promising complementary, non-invasive testing strategy—particularly for unreached or underscreened populations—rather than as a replacement for established screening pathways (27).

The feasibility of FMU collection also warrants candid appraisal. Of 638 women recruited, 138 (21.6%) were excluded because their specimens did not meet the standardized quality criteria—most commonly nocturnal voiding precluding a true first-morning sample (n=50), failure to capture the first-void fraction (n=35), transport time exceeding 2 hours (n=25), improper storage temperature (n=18), and leakage during transport (n=10). This non-trivial failure rate, and in particular the requirement for specimen transport within 2 hours, may be difficult to meet in geographically remote or resource-limited regions and represents a practical barrier to scale-up. Future work should evaluate whether less stringent collection rules are feasible—for example, urine stabilizing/preservative buffers (such as EDTA-based or commercial conservation media) to extend the permissible transport interval, dried-urine or FTA-card formats that tolerate ambient transport, and home mailing of self-collected specimens—so that diagnostic performance can be maintained while reducing the specimen-failure rate and broadening real-world applicability.

Several limitations warrant acknowledgment. First, this was a disease-enriched, largely hospital- and colposcopy-based cohort rather than an average-risk screening population; the enriched CIN2+ prevalence (40%) inflates the PPV and limits direct generalizability of the predictive values to routine population-based screening, where performance remains to be validated. Second, the study employed a partial verification design: only participants with abnormal co-testing underwent colposcopy and biopsy, whereas women with negative co-testing were classified as no-lesion without histological confirmation. This may introduce verification (work-up) bias, which characteristically over-estimates sensitivity and under-estimates specificity for both specimen types; the absolute accuracy estimates should therefore be interpreted with this directional bias in mind. Third, tissue-based HPV genotyping of biopsy specimens was not performed, so for CIN2+ cases negative on both liquid specimens we could not distinguish truly HPV-independent lesions from false-negative sampling. Fourth, the standardized collection protocol yielded a 21.6% specimen-failure rate, and the 2-hour transport requirement may limit applicability in remote settings (discussed above). Fifth, the modest specificity of both HPV testing modalities (approximately 64–67%) could be improved by integration with reflex biomarkers such as HPV E6/E7 mRNA or p16INK4a/Ki-67 dual-stain immunocytochemistry (28), an avenue warranting future investigation. Sixth, the single-region design and 8-month recruitment window preclude assessment of seasonal variation in specimen quality and longitudinal outcome follow-up. Finally, formal cost-effectiveness analysis and postal self-collection feasibility studies are needed before policy-level implementation recommendations can be issued (19, 27).

## Conclusions

First-morning urine (FMU) exfoliated cell HPV testing demonstrated diagnostic accuracy comparable to traditional cervical HPV testing for CIN2+ detection in this disease-enriched diagnostic cohort (sensitivity 90.50%, specificity 67.00%, PPV 64.64%, NPV 91.36%). Because no predefined non-inferiority margin was tested and the cohort was enriched for high-grade disease, these estimates—particularly the predictive values—should not be extrapolated to average-risk, population-based screening without further validation. Its non-invasive self-collection design, combined with significantly superior participant compliance (97.20% vs 84.80%) and satisfaction (95.40% vs 79.60%), positions FMU HPV testing as a promising complementary, non-invasive testing strategy. Pending validation in low-risk screening populations and optimization of specimen-collection logistics, implementation within structured cervical cancer screening programs—particularly in regions with geographic, cultural, or logistical barriers—has the potential to increase screening coverage and reduce cervical cancer incidence and mortality.

## Data Availability

The original contributions presented in the study are included in the article/supplementary material. Further inquiries can be directed to the corresponding authors.
